# Investigations on the effect of natural veined calcite on the mechanical properties of limestone

**DOI:** 10.1038/s41598-024-56068-4

**Published:** 2024-03-11

**Authors:** Qingzhi Chen, Yuanming Liu, Zhaolei Teng, Xun Ou, Quan Zhang

**Affiliations:** College of Civil Engineering, Guizhou University, Guiyang, 550025 Guizhou China

**Keywords:** Calcite veins, Acoustic emission, Digital image correlation, Uniaxial compression test, Civil engineering, Mechanical properties

## Abstract

The damage behavior of limestone rock masses containing calcite mineral filling under uniaxial compression experimental conditions is unclear, and the fracture mechanism of the rock masses needs to be further explored. In this study, uniaxial compression tests were conducted on limestone rock specimens containing veined calcite by combining acoustic emission and digital image correlation techniques. The effects of veined calcite on the generation and development of cracks on the surface of the specimens until the formation of macroscopic penetration and the strength properties of the rock mass were analyzed. The results showed that the transversely distributed veined calcite caused significant stress concentrations in the rock specimens. The longitudinally distributed veined calcite caused cracks in the specimens or influenced the expansion path of the longitudinal principal cracks. The final damage pattern of the specimens didn’t differ significantly from that of conventional rock masses due to the presence of veined calcite. The presence of the veined calcite had effect on the uniaxial compressive capacity of the rock, but the load variation process of the specimen with time still conformed to the load variation pattern during the uniaxial compressive test of conventional rocks.

## Introduction

Mineral veins play a crucial role in various geological contexts^1,2^. They are nearly ubiquitous in rocks and are readily identifiable in the field due to the distinct color contrast between vein minerals and their host rocks. The mechanical properties of veins typically differ significantly from those of the host rock, resulting in discontinuities in the mechanical properties of rock masses^1–4^. This divergence facilitates the influence of veins on the expansion of internal cracks when rock masses are subjected to stress, thereby affecting the fracture behavior of the rock. Consequently, the geomechanical behavior of veins holds paramount importance in numerous rock engineering projects^5–7^. Comprehending the impact of veins on the mechanical behavior of rocks is essential for the rock engineering design of veined rock masses. Insufficient consideration of veins in rock engineering design may lead to inaccurate predictions of rock behavior, potentially resulting in substantial economic losses and heightened safety risks^7–9^. While prior studies have predominantly focused on the formation of calcite veins, delving into aspects such as physical and microstructural features or geochemistry, limited attention has been given to understanding the effect of calcite veins on the mechanical properties of rocks. For instance, Bons et al.^10^ provided an overview of the main facets of vein formation, ranging from fracture mechanics to petrology and geochemistry. Additionally, Steen et al.^11^ investigated the influence of calcite veins on the fracture behavior of limestone using petrographic microscopy, scanning electron microscopy, and reflection microscopy.

Based on prior research on the mechanical properties of rocks, it is evident that a majority of studies have focused on homogeneous rock specimens comprising a single type, such as pure mudstone, sandstone, or limestone. There has been comparatively less exploration into the mechanical properties of more intricate rock specimens, such as limestone rock masses with calcite veins. Moreover, the existing studies on rock damage primarily center on investigating the strength characteristics of the rock mass concerning fractures^12–16^. Nevertheless, the mechanical properties of rock masses in natural environments are intricately linked not only to factors like fractures but also to the inherent complexity of the rock mass itself. In the context of this study, the amalgamation of rocks with diverse lithological types within a rock body, along with the combination of veins (calcite) with the primary rock body (limestone), may potentially impact the overall mechanical properties of the rock masses. Given the widespread distribution of rocks containing vein fillers in the project's rock masses, concerns arise regarding whether their presence could adversely affect the structural integrity, stability, and strength properties of the tunnel's surrounding rocks.

The abundance of these vein fillers may significantly alter the path of crack extension in the surrounding rock during the excavation process, deviating from that observed in intact and lithologically uniform rock masses. This introduces uncertainty into the tunnel excavation process, escalating the difficulty, danger, and unpredictability of the surrounding rock's rupture pattern, ultimately impeding the safe and efficient excavation of the tunnel. Therefore, a comprehensive study on the influence of veins on the mechanical properties of rocks becomes imperative. Considering these factors, this study, grounded in previous relevant research and contextualized within actual engineering scenarios, selected natural rock masses containing conspicuous calcite veins as specimens from a partially excavated tunnel section. The objective is to investigate the impact of the presence of vein fillers on the mechanical properties and damage characteristics of the rock.

Acoustic Emission (AE) testing serves as a potent tool for investigating the propagation of defects in brittle materials such as rocks. The strength or frequency of AE events can effectively signify the intensity of microcracking and its progression within the rock. Researchers routinely delve into the fracture characteristics and processes of rocks by examining AE parameters, including the number of AE events, amplitude and frequency distribution, and energy^17–19^. Presently, AE techniques find widespread application in the examination of damage and fracture behavior within rock materials. For instance, Jiang et al.^20^ explored and explicated the correlation between the size of rock cracks and the frequency characteristics of AE signals induced during tensile fracture through indirect tensile tests. Zha et al.^21^ conducted a triaxial creep test with step loading on deep marble, monitoring AE characteristic parameters throughout extended periods of creep. Their study revealed that variations in AE parameters during specific stages of the creep test could distinguish the initial creep stage, steady-state creep stage, and accelerated creep stage of the rock.

Digital Image Correlation (DIC) stands out as an optical, non-contact deformation measurement technique employed to determine the spatial distribution of displacement and strain in an object during deformation. In comparison with alternative deformation measurement methods, this technique boasts distinct advantages: lower environmental requirements, a straightforward testing apparatus, and user-friendly operation. The application of DIC techniques in researching crack extension, penetration, and damage and fracture behavior on the surfaces of materials, such as rock masses or rocks, has become a prevalent avenue of investigation^22–25^. Lin et al.^22,23^ delved into the crack coalescence process of specimens with joints under uniaxial loading using AE and DIC techniques. Niu et al.^24^, utilizing AE-monitored b-value analysis, examined the time-varying damage evolution of fracture-containing rocks subjected to uniaxial compression. Concurrently, the DIC technique captured the real-time cracking process of rocks containing fractures. As illustrated, both the AE technique and DIC are firmly established in the field of rock mechanics, offering effective methodologies and convenient conditions for advancing the study of rock mechanics.

Given the challenges and cost implications associated with obtaining natural rock specimens, scholars frequently resort to simulating natural rock masses by artificially introducing single or multiple joints. Infrequently, rock specimens with natural joints, fractures, or fillers are utilized to analyze the mechanical properties of rock masses^26–28^. Consequently, studies based on artificially created rock-like materials have inherent limitations. While these investigations aptly highlight the impact of individual structural elements, such as joint undulation angle and connectivity, on the mechanical properties of rock masses, it's essential to note that the distribution of structural facets or vein fills in these artificially produced specimens tends to be regular and more uniformly arranged. This is in stark contrast to the natural distribution of structural facets or vein fills within rock masses in authentic environments. As a result, such studies fall short of fully capturing the mechanical and damage properties of rock masses containing irregular structural facets or filled materials in natural settings.

Rocks containing vein fillers are prevalent in the project's rock masses, prompting concerns about their potential adverse effects on overall structural integrity, stability, and strength properties of the tunnel's surrounding rocks. The substantial presence of these vein fillers may significantly alter the path of crack extension in the surrounding rock during the excavation process compared to that in an intact and lithologically uniform rock mass. This introduces uncertainty to the tunnel excavation process, elevating the difficulty, hazards, and unpredictability of surrounding rock rupture. Such uncertainties pose challenges to the safe and efficient excavation of the tunnel. Therefore, it is imperative to conduct a comprehensive study on the impact of veins on the mechanical properties of rocks.

In this study, with a foundation built upon previous pertinent research and a backdrop of real-world engineering scenarios, natural rock masses featuring distinct calcite veins were selected as specimens from the partially excavated section of the tunnel. The objective was to scrutinize the impact of calcite vein fillers on the mechanical properties and damage characteristics of the rock. Considering the drawbacks of utilizing rock-based materials for laboratory experiments and acknowledging the actual conditions of natural rocks in the project under consideration, uniaxial compression tests were conducted. These tests were complemented by AE and Two-Dimensional DIC techniques to explore the strength characteristics of rock masses containing calcite fillings, as well as the influence of the original calcite veins on the overall crack penetration characteristics of the specimens.

## Preparation of specimens and test methods

### Engineering background

**Figure 1:**
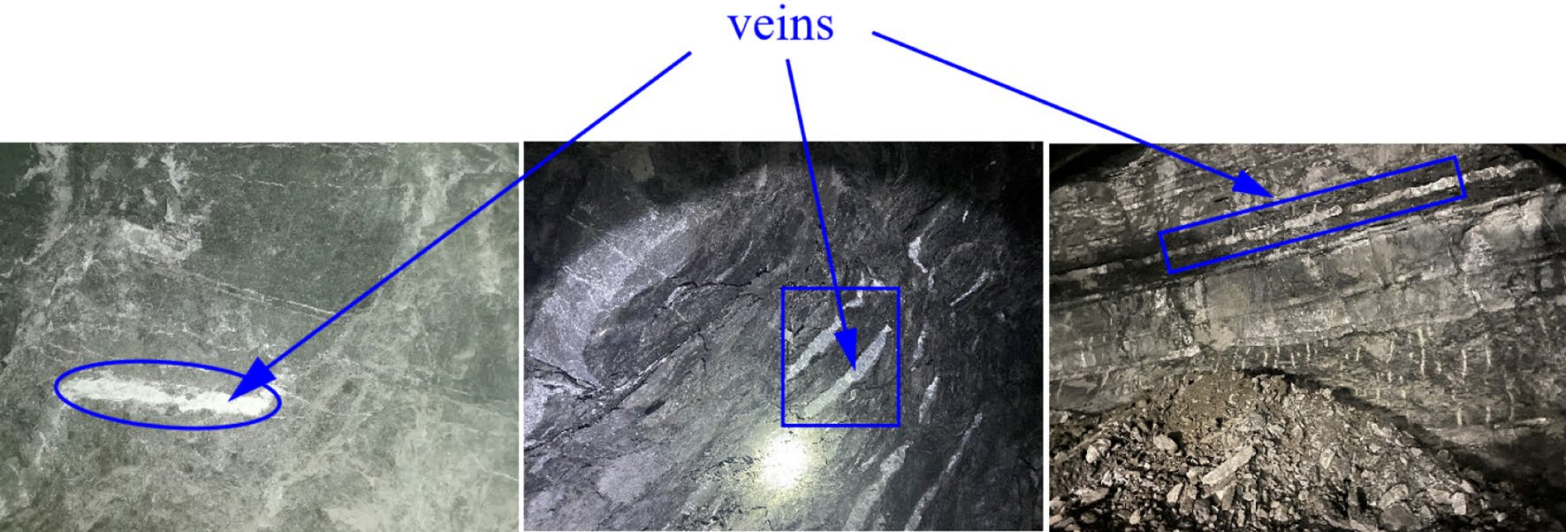
The actual conditions of the surrounding rock.

In the encompassing rock formations of the tunnel under investigation, geological characteristics exhibit heightened complexity, featuring prevalent rock types such as siltstone, mudstone, muddy siltstone, limestone, and muddy limestone. Siltstone, mudstone, and muddy siltstone manifest in shades of gray, gray-yellow, and gray-green, presenting a laminated structure with smaller thickness. These rocks are characterized by well-developed joints and fissures, contributing to their fragmented nature. Limestone, on the other hand, appears in varying shades of gray, light gray, and dark gray, showcasing a thicker laminated structure with less pronounced joints within the rock body. Consequently, limestone rocks exhibit greater structural integrity and hardness. Muddy limestone, identified by its gray and dark gray hues, is characterized by a thin-layered structure and a softer rock quality. However, within a defined region of the tunnel perimeter, alongside the aforementioned lithologies, limestone rock masses containing calcite vein fillings are frequently encountered, as depicted in Fig. 1.

### Preparation of specimens

**Figure 2:**
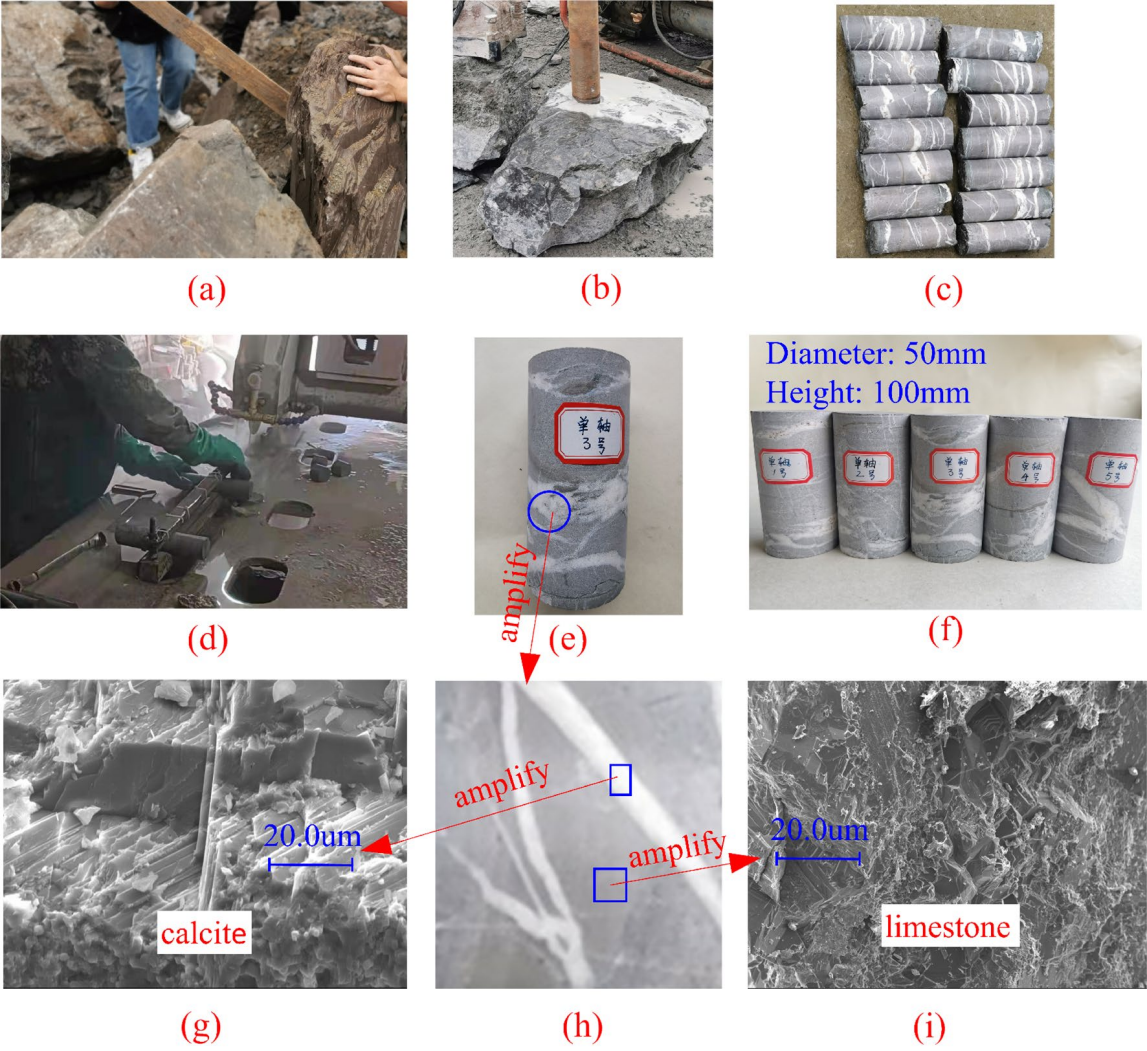
The process of specimen preparation and determination of its composition: (**a**) Selection of rock blocks, (**b**) Drilling of rock cores, (**c**) The calcite veins presented on the surface of the cores, (**d**) Polishing of specimens, (**e**) and (**f**) Demonstration of the finished specimen, (**g**) Microstructure demonstration of limestone, (**h**) Enlarged view of calcite veins, (**i**) Microstructure of calcite fillings showing.

The specimen preparation process is depicted in Fig. 2. In the excavated sections of the ZK42 + 856 and YK42 + 666 tunnels, sizable and well-preserved rock bodies containing evident fill were meticulously chosen, and cores were drilled, as illustrated in Fig. 2a, b. It's imperative to note that all cores should originate from the same block, ensuring uniform composition across all specimens. Given that subsequent tests primarily involved uniaxial compression on intact, small-sized specimens containing veins, the potential influence of the damage zone around the tunnel wall was not factored into the selection of the rock mass.

The veins evident on the core surfaces are highlighted in Fig. 2c. Subsequently, the cores underwent polishing to yield the specimens required for subsequent uniaxial compression tests, as demonstrated in Fig. 2d. The resulting specimens are showcased in Fig. 2e, f. This study utilized five specimens, each with a diameter of 50 mm and a height of 100 mm. Electron microscope scanning images of the two compositions are presented in Fig. 2g, i.

From Fig. 2g, i, a notable disparity in microstructure between the veins and the host rock is evident. Consequently, there is a high probability that they possess distinct mechanical properties, suggesting the veins may significantly impact the overall mechanical properties of the rock. This underscores the imperative to investigate the mechanical properties of rock masses containing veins. Furthermore, the color and major elements of both the veins and the host rock (as detailed in Table 1) indicate that the veins consist of calcite, while the host rock is limestone.

**Table 1:** The major elements contained in veins and the host rock.

	Major elements (atomic percentage %)									
	C	O	Na	Mg	Al	Si	S	K	Ca	Fe
Veins	31.41	40.63	0.09	0.03	0.56	8.50	0.58	0.01	18.15	0.03
Main rock	25.58	43.69	1.12	0.09	1.12	9.21	0.35	0.04	18.73	0.07

To investigate the influence of calcite-filled veins on crack extension and the strength properties of rock specimens during uniaxial compression tests, specific specimens earmarked for these tests underwent color highlighting and marking. Calcite veins entirely filled with calcite were delineated with a red marker, while calcite veins displaying incomplete filling and gaps were marked with a blue one. This distinction aimed to accentuate the calcite vein fillings, allowing for enhanced observation and analysis using the 2D DIC digital scatter system. The natural state of the specimens before testing and their condition post-artificial accentuation of calcite veins is detailed in Table 1. Additionally, physical properties such as density and wave speed for each specimen are presented in Table 2.

**Table 2:** Details of specimens.

	Number of each specimen				
	JL1	JL2	JL3	JL4	JL5
Original state of the specimens					
Specimens after prominent calcite veins					
Density (kN/m^3^)	25.95	25.97	26.04	26.07	26.02
Wave speed (m/s)	5628	5631	5654	5673	5597
Average diameter (mm)	50				
Average height (mm)	100				

### Test method

**Figure 3:**
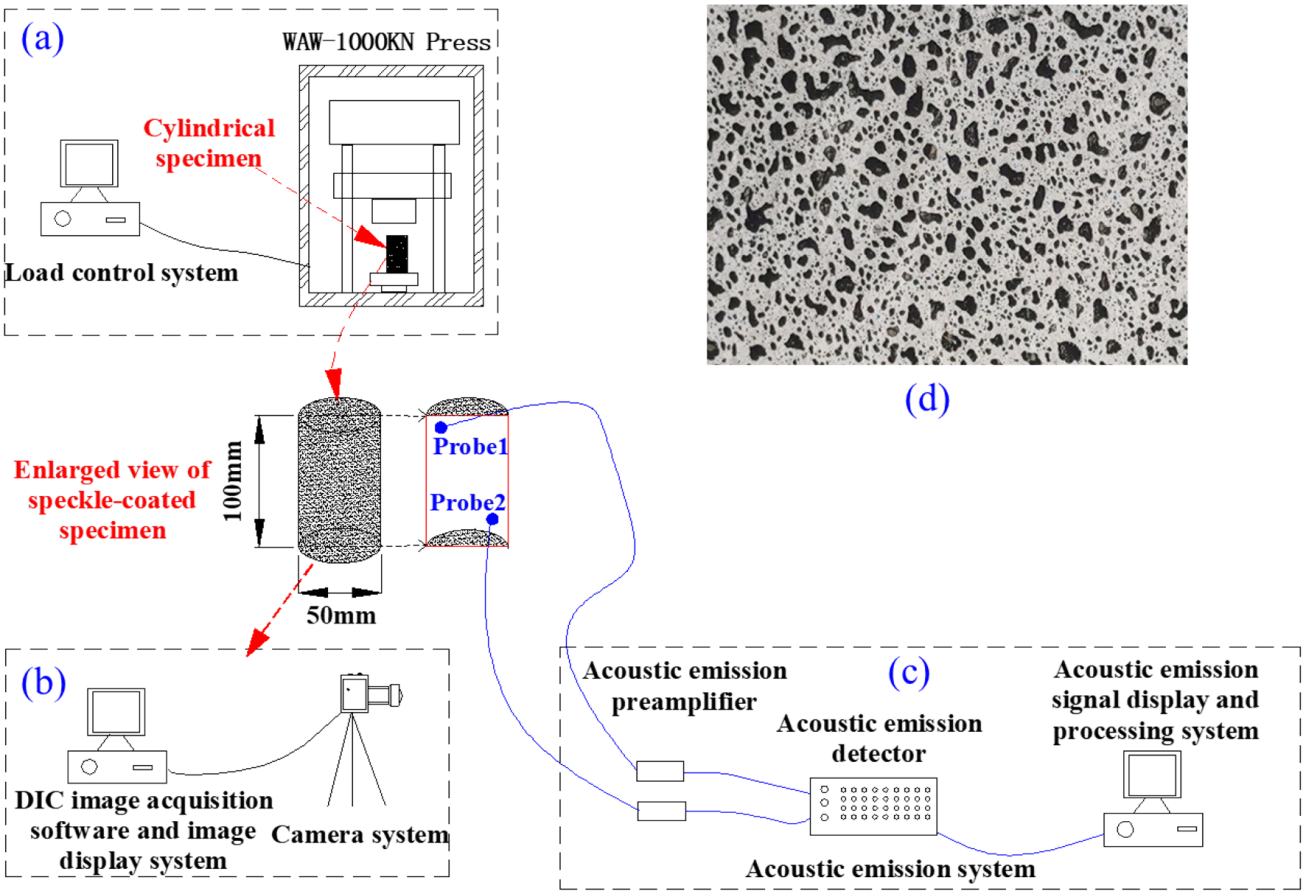
Schematic diagram of the device used for uniaxial compression test.

**Figure 4:**
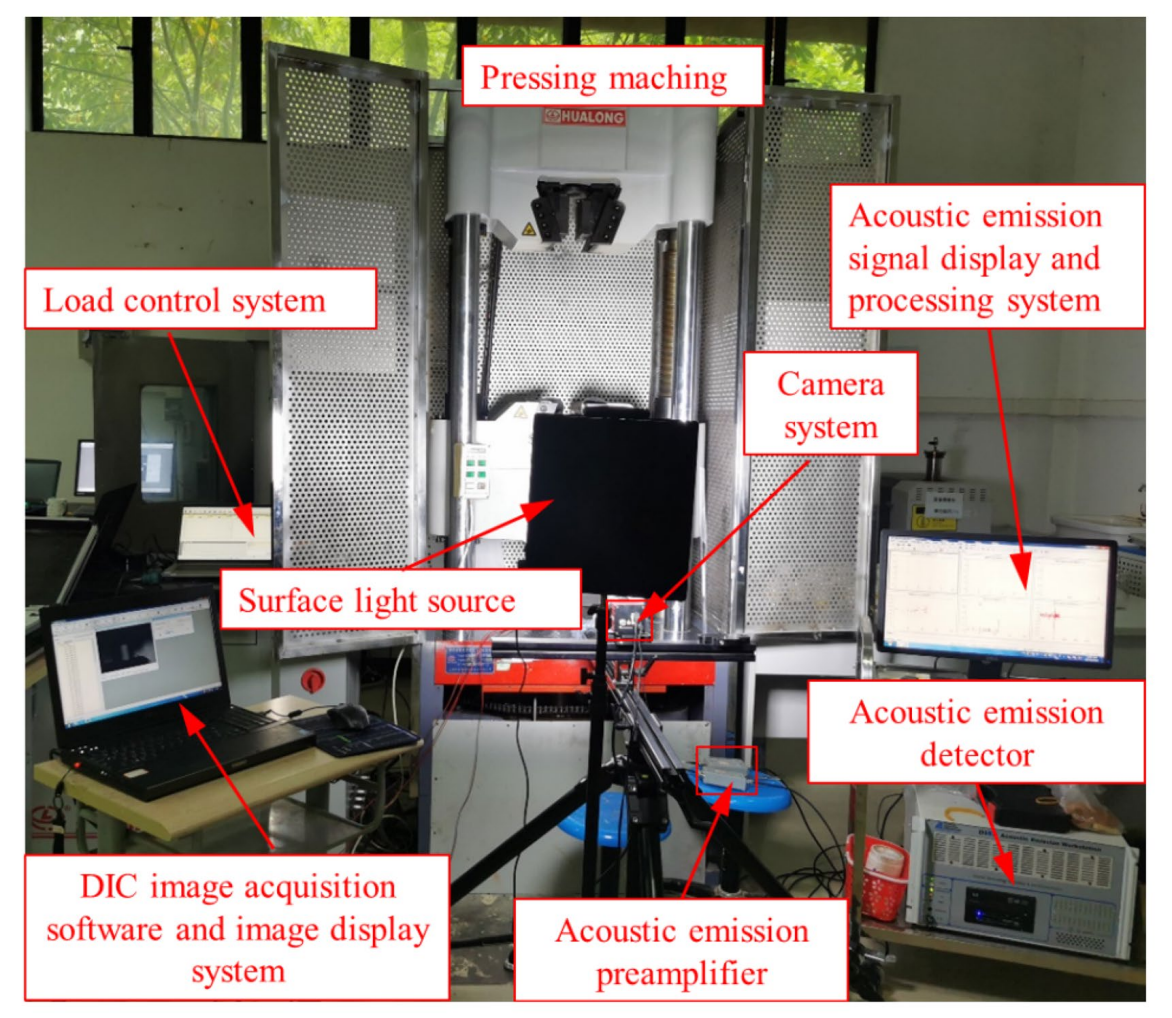
Field diagram of the device used for uniaxial compression test.

In this study, uniaxial compression tests were conducted on limestone specimens containing calcite veins using a WAW-1000KN microcomputer-controlled electro-hydraulic servo universal testing machine. This approach allowed for a comprehensive exploration of the rock mass's strength characteristics, while concurrently observing the state of crack development and damage patterns in the specimens through the application of AE (PCI-2) and DIC techniques (GE4900). The installation arrangement of all testing equipment is delineated in Figs. 3 and 4.

During the uniaxial compression test, the loading rate was controlled by managing longitudinal displacement, with a preloading rate set at 5mm/min and a formal uniaxial test loading rate of 1 mm/min. Upon the axial load dropping to 20% of the maximum force, the specimen was deemed to have reached a damaged state, prompting automatic cessation of loading by the instrument. Alternatively, manual control could directly end the loading when deemed suitable to terminate the test. To enhance experimental data processing and control, the loading system, AE system, strain measurement system, and high-speed camera system were manually toggled on and off simultaneously.

The study employed a combined approach of AE and 2D DIC techniques during the uniaxial compression test to capture data and information, including rock time-axis force curves, AE event numbers, and 2D DIC digital analysis images throughout the rock damage process. This comprehensive dataset facilitated the observation of new crack propagation paths during the experiment and allowed for an analysis of the impact of original calcite veins on crack expansion, damage patterns, and strength characteristics of the specimens.

Prior to the test, the specimen's surface is coated with a scattering material meeting the requirements of the DIC analysis technique. To create artificial scattering on the specimen's surface, a thin and uniform layer of white paint is applied. After the white paint dries, black paint is sprayed randomly from approximately 30 cm above the specimen. The black paint particles naturally descend onto the white base, forming randomly distributed artificial spots. It is crucial to note that the black spots should exhibit randomness, uniformity, and moderate density. The intricate details of the final specimen surface, showcasing these artificial spots, are presented in Fig. 3d.

Throughout the test, the high-speed camera system is conFig.d to capture images at a rate of 3 frames per second, providing a real-time representation of the specimen's status during the entire loading process. Positioned approximately 1 m away from the specimen, the camera system utilizes two surface light sources as its illumination system, ensuring a stable and reliable light source. This setup enables the camera system to capture high-quality, high-definition image information.

For AE monitoring, two probes are employed for signal acquisition, with their arrangement illustrated in Fig. 3. Vaseline serves as a coupling agent, securing the probes tightly to the specimen's surface to maximize AE signal acquisition. The AE monitoring system is conFig.d with a pre-gain of 40 dB, a threshold value of 40 dB to mitigate external ambient noise interference, and a sampling rate of 1 MSPS.

### Informed consent

The datasets used and/or analyzed during the current study are available from the corresponding author on reasonable request.

## Test results and analysis

### The influence of calcite veins on the crack extension process and damage mode of specimens

Throughout the uniaxial compression test, the application of DIC technology, coupled with high-speed camera equipment, offers a more direct visualization of the initiation, development, and macroscopic through-crack formation process in the form of images. In this study, the 2D DIC system was utilized to analyze the variation of principal strain at the observed surface of each specimen throughout the entire experiment.

**Figure 5:**
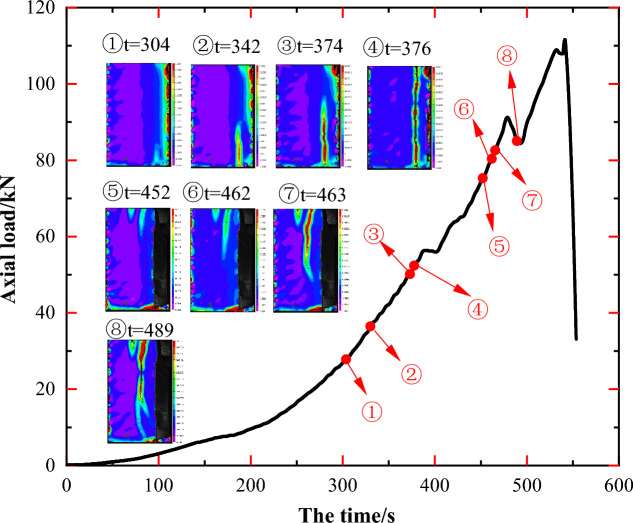
Principal strain state at some moments on the surface of JL1 specimen.

Comparisons of principal strain images at various time points provide a more intuitive depiction of changes in principal strain at each point on the specimen surface over time. This approach highlights the location of stress concentration regions in the specimen, the detailed progression of crack generation, development, and penetration, as well as the impact of the original rock mass structure on the resultant damage pattern and the expansion path of newly formed cracks. As illustrated in Figs. 5, 7, 9, 11 and 13, markers ①–⑧ represent principal strain states on the specimen surface at specific time points for each specimen. These markers effectively outline key information about stress concentration, major crack initiation, and final specimen damage throughout the entire experiment.

It's noteworthy that the 2D DIC system cannot provide information within a 6 mm width at the top and bottom of the specimen. This limitation arises due to the use of two rubber sleeves to secure the AE probes at the top and bottom of the specimen during the experiment.

**Figure 6:**
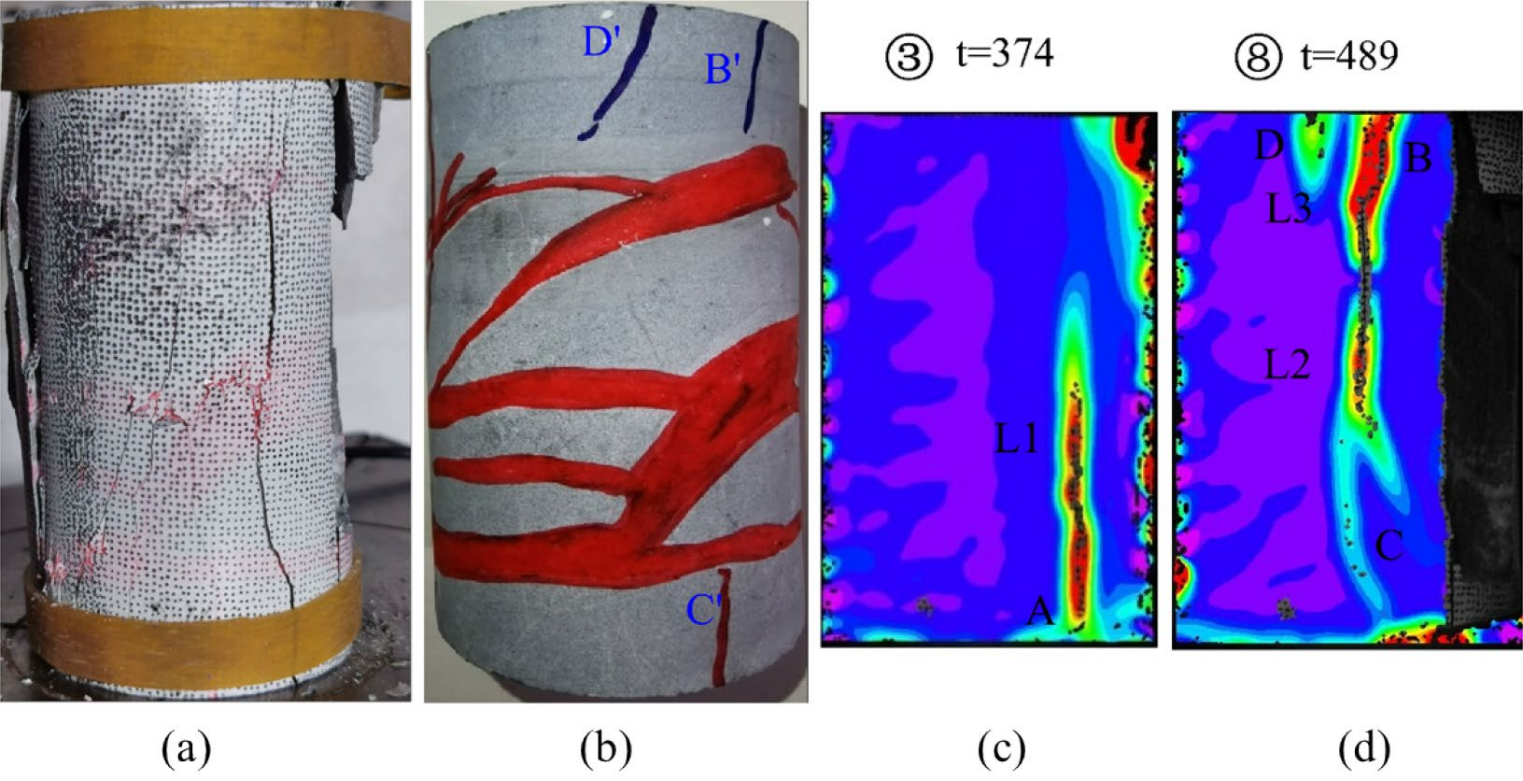
Analysis of the state of the JL1 specimen before and after the test and the main strain during the test: (**a**) Final state of the specimen, (**b**) Initial state of the specimen, (**c**) Principal strain of the specimen at t = 374 s, (**d**) Principal strain of the specimen at t = 489 s.

Figure 6 presents photographs depicting the initial and final states of specimen JL1, along with the corresponding 2D DIC principal strain images at t = 374 s and t = 489 s. Combining markers ①–⑧ from Fig. 5, it becomes evident that a microcrack emerged along the longitudinal distribution in part A of the specimen during steps ①–⑤. This microcrack evolved into the through main crack L1, extending longitudinally from the top to the bottom of the specimen (as depicted in Fig. 6c). When the maximum principal strain of crack L1 reached approximately 0.05 mm, longitudinal splitting damage occurred on the right side of the specimen, leading to stress redistribution in the orthogonal plane. Subsequently, during steps ⑥–⑧, two longitudinally distributed cracks, L2 and L3, were formed along the right side of the remaining part of the specimen (as shown in Fig. 6d). The crack L2 continued to expand along the longitudinal direction as the load was applied, nearly achieving full penetration when its maximum principal strain reached 0.043 mm.

Upon comparing the pre-experimental and post-damage photographs of the specimens in Fig. 8, and examining the main strain images at t = 374 s and t = 489 s, it was observed that the initiation point B and penetration point C of the aforementioned main fracture L2 partially coincided with the positions of the primary veins calcite B' and C' of the specimen depicted in Fig. 6b.

Hence, the strain and crack development throughout the entire uniaxial compression test of specimen JL1 can be delineated into two distinct processes: the evolution and penetration of the longitudinal crack L1, and the initiation and expansion of cracks L2 and L3. Notably, the extension and penetration processes of these cracks exhibit a significant correlation with the presence of primary calcite veins. The primary crack, L2, demonstrates an extension direction, initiation, and termination that coincide with the locations of primary calcite veins. Consequently, the specimen underwent damage consistent with a splitting pattern under the conditions of the uniaxial compression test.

**Figure 7:**
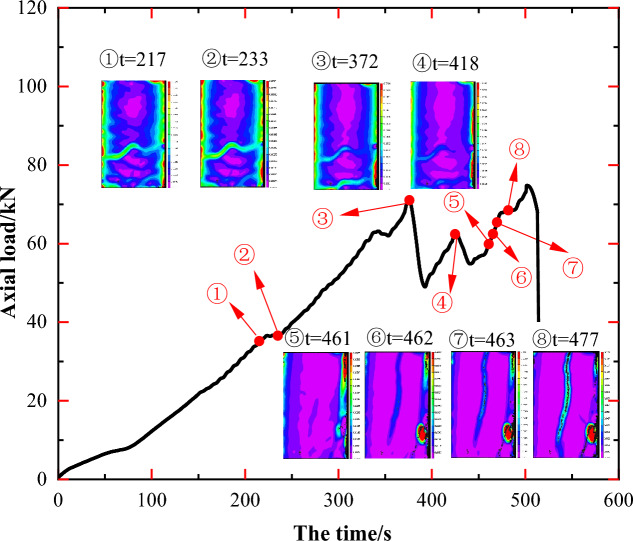
Principal strain state at some moments on the surface of JL2 specimen.

**Figure 8:**
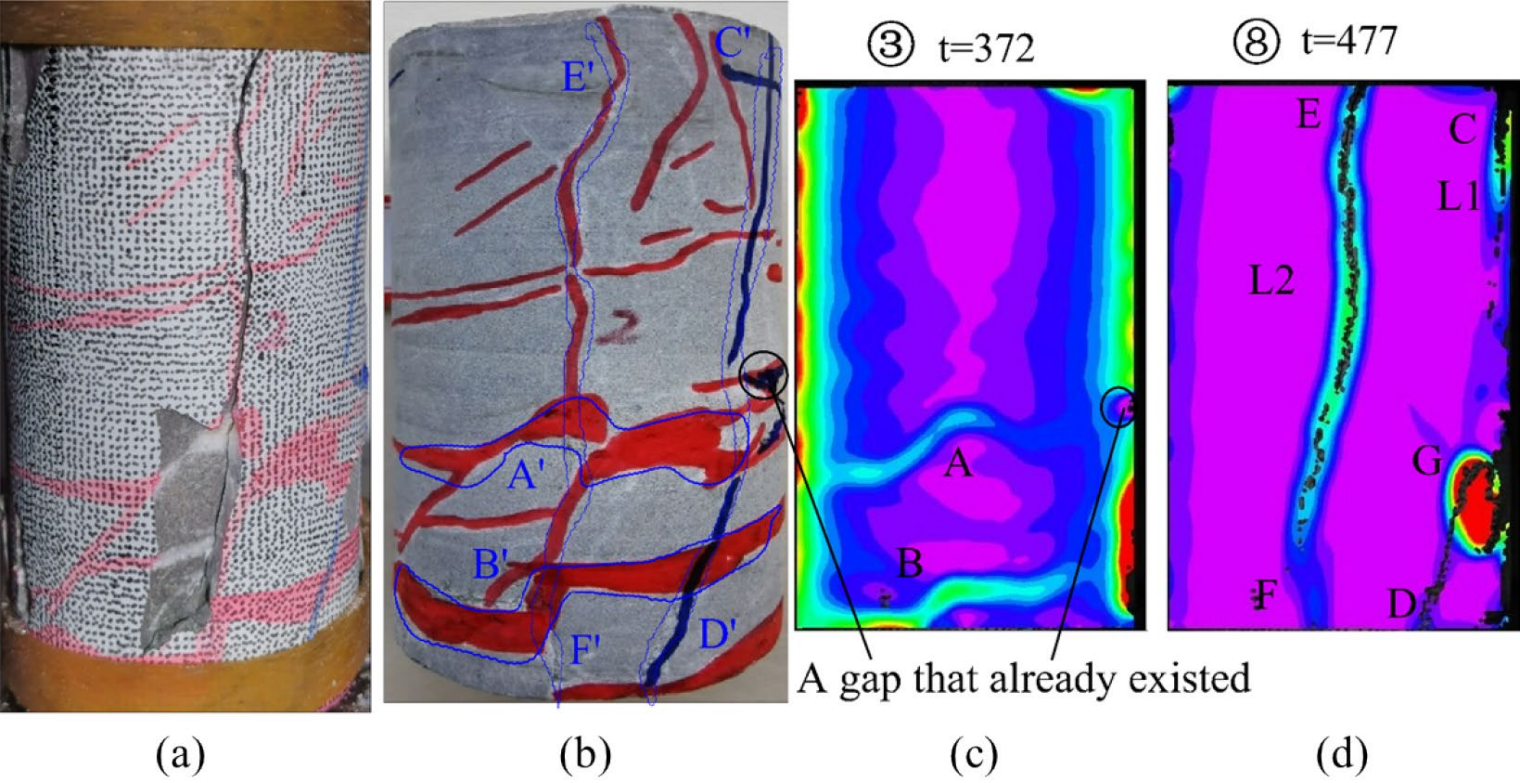
Analysis of the state of the JL2 specimen before and after the test and the main strain during the test: (**a**) Final state of the specimen, (**b**) Initial state of the specimen, (**c**) Principal strain of the specimen at t = 372 s, (**d**) Principal strain of the specimen at t = 477 s.

Figure 8 presents pre- and post-test photographs of specimen JL2 along with main strain images at t = 372 s and t = 477 s. Examining the principal strain states of specimen JL2 at different times (①–⑧) in Fig. 7, it is discerned that the principal strain value near the stress concentration region A, as depicted in Fig. 8c, increases from state ① (0.0032–0.0058) to state ③ (0.0065–0.0092), then decreases to state ⑤ (0.0025–0.0065) from ① to ⑤. The principal strain value at area B exhibits a similar pattern of increase and subsequent decrease. This suggests that although stress concentration and transverse penetration occurred at regions A and B during steps ①–⑤, no new cracks developed in the stress-concentrated region as the experiment progressed. Ultimately, stress redistribution led to the disappearance of stress concentration at region A.

Upon comparing the state of the specimen before the test in Fig. 8b with the principal strain image at t = 372 s in Fig. 8c, it becomes apparent that stress concentration areas at A and B in the 2D DIC image correspond to calcite veins A' and B' in the original specimen, respectively. This indicates that during the uniaxial compression test of JL2, calcite veins A' and B' within the specimen influenced the stress distribution of the rock mass. However, they lacked sufficient capability to induce new cracks in the specimen.

After the stress redistribution process at the midpoint of the specimen during steps ①–⑤, as the axial load increased, the specimen exhibited stress concentration at position C, as depicted in Fig. 8d during steps ⑤–⑥. The stress concentration area gradually extended from C to D, giving rise to the development of the main crack L1 from top to bottom, accompanied by a distinct stress concentration area at G. A comparative analysis of the specimen states in Fig. 8a, b and d reveals that the penetration path CD of the main crack L1 overlaps with the original calcite veins C'D', and the location of the stress concentration region G coincides with the right end of the original calcite veins B'.

Subsequently, during the steps depicted in ⑥–⑧, the specimen generated a new crack at E after the penetration of the main crack L1. This crack propagated from point E to point F, resulting in the longitudinal penetration of the main crack L2 (as shown in Fig. 8d), ultimately leading to the specimen's final damage. A comparison of the expansion path of the main crack L2 with the distribution of the original calcite veins in the specimen reveals a more extended overlap with the original calcite veins E'F' in Fig. 8b.

In summary, throughout the damage progression of specimen JL2, a clear alignment emerges among regions exhibiting more prominent main strain changes in the 2D DIC images, the penetration paths of newly generated cracks L1 and L2, and the distribution regions of the original calcite veins within the specimen. This signifies a notable influence of the original calcite veins on the development of new cracks in specimen JL2 during the experimental process.

Concerning specimens JL1 and JL2, a consistent observation is evident, indicating that cracks consistently exhibit a proclivity to traverse areas of inherent weakness. Within these specimens, calcite-filled veins act as pathways, creating zones of vulnerability. As a result, stress concentration manifests at these specific planes, concurrent with the initiation of cracking.

**Figure 9:**
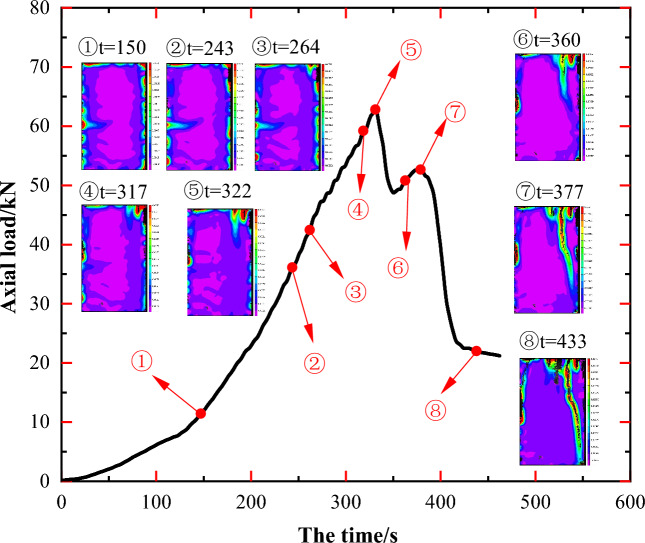
Principal strain state at some moments on the surface of JL3 specimen.

**Figure 10:**
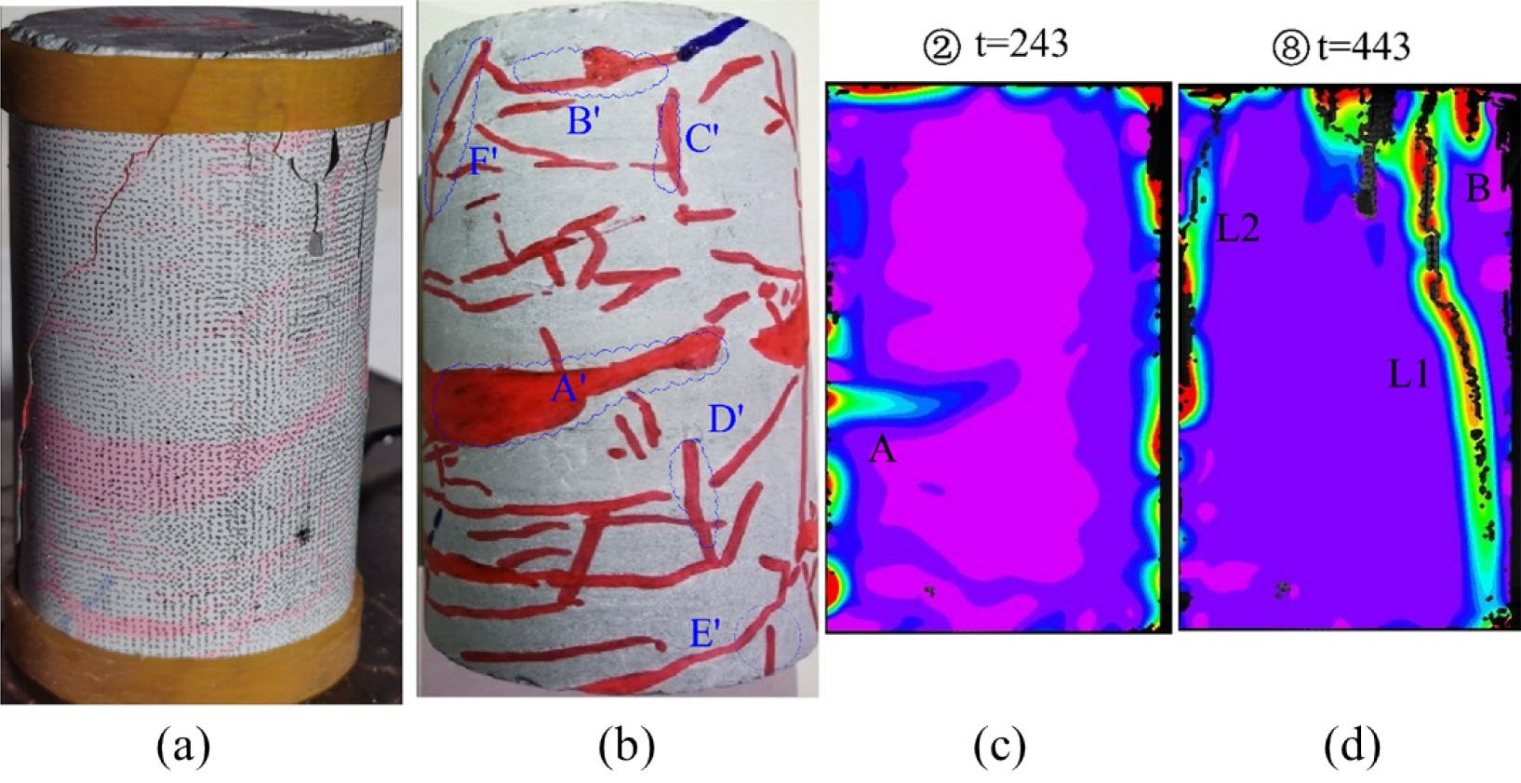
Analysis of the state of the JL3 specimen before and after the test and the main strain during the test: (**a**) Final state of the specimen, (**b**) Initial state of the specimen, (**c**) Principal strain of the specimen at t = 243 s, (**d**) Principal strain of the specimen at t = 443 s.

Upon analyzing the principal strain image of specimen JL3 in Fig. 9, a resemblance to specimen JL2 becomes apparent. The central region A of specimen JL3 (as depicted in Fig. 10b) also experienced transverse stress concentration during steps ①–④, gradually diminishing as the experiment progressed. A comparative examination of the specimen states in Fig. 10b, c reveals a more pronounced overlap of region A with the position and orientation of calcite veins A' in the original specimen. This implies that although the calcite veins did not induce a conspicuous crack in specimen JL3 at this position, they did result in a noticeable stress concentration region in the specimen at this specific location.

During steps ④–⑧, multiple stress concentration points appear in the upper right side of the specimen at B, where new cracks begin to develop. Eventually, a more prominent and primary crack, L1, emerges, extending up and down along the axial direction of the specimen within area B. A comparison between Fig. 10b, d reveals that stress concentration region B corresponds to the region B' where the original calcite veins are distributed in the specimen. The penetration path of the main crack L1 coincides with the calcite veins at C', D', and E'. This suggests a direct influence of the original calcite veins on the initiation and penetration path of the main crack.

Furthermore, during the penetration of the main crack L1, an additional penetrating crack, L2, originates from the middle to the top of the left side of the specimen. Upon comparing Fig. 10a, b and d, it is evident that the initiation position of this crack aligns with the starting position of calcite veins A', and the overall expansion path of crack L2 precisely coincides with the original calcite veins F'. A comprehensive analysis of the entire damage process of specimen JL3 reveals four main stages: stress concentration in the middle lateral part, stress redistribution, emergence and penetration of crack L1, and emergence and penetration of crack L2. Throughout the process, the emergence and extension of cracks are notably influenced by the presence of primary calcite veins.

**Figure 11:**
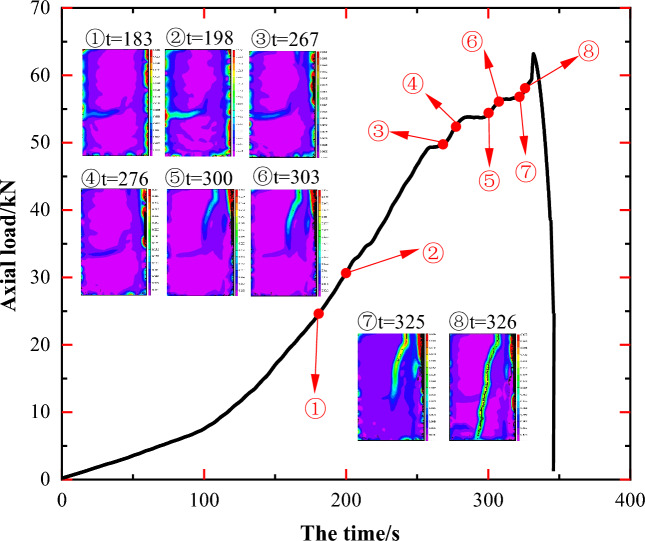
Principal strain state at some moments on the surface of JL4 specimen.

**Figure 12:**
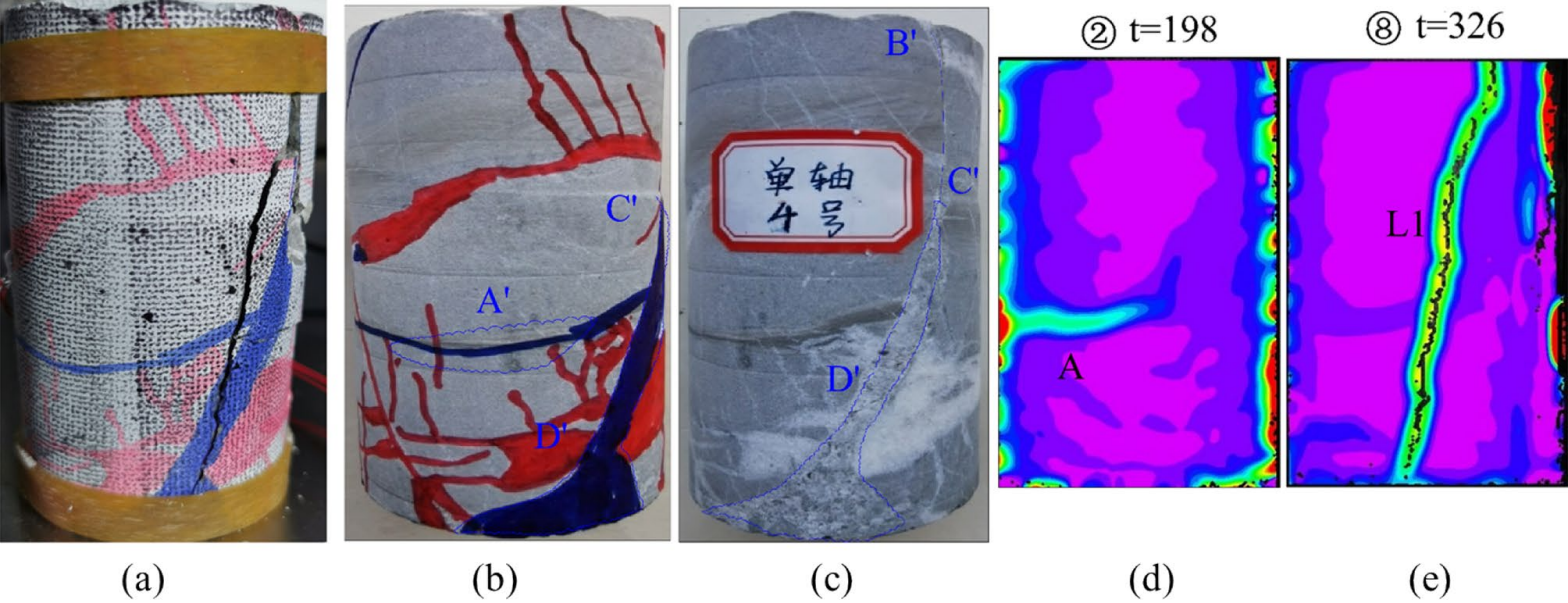
Analysis of the state of the JL4 specimen before and after the test and the main strain during the test: (**a**) Final state of the specimen, (**b**) and (**c**) Initial state of the specimen, (**d**) Principal strain of the specimen at t = 198 s, (**e**) Principal strain of the specimen at t = 326 s.

The image of JL4 in Fig. 11 reveals that, akin to specimens JL2 and JL3, a stress concentration and diffusion process occurred in the middle of specimen JL4. Upon analyzing the image information in Fig. 12, it is evident that the region of stress concentration, denoted as A, coincides with the location of the calcite veins A' in the specimen as shown in Fig. 12b. A comparative examination of the location where the main crack L1 initiates and the path of its expansion with the final damage state of the specimen in Fig. 12a indicates that the location of the crack L1 initiation aligns with the end point B' of the native calcite veins depicted in Fig. 12c. Furthermore, its path of expansion coincides with B'C' and ultimately traverses through D' within the interior of the calcite veins.

In summarizing the crack expansion process of specimen JL4, it becomes apparent that the stress concentration, as well as the initiation and expansion location of the main crack L1, align more closely with the position of the original calcite veins. Consequently, the crack expansion of specimen JL4 was notably influenced by the presence of calcite veins.

**Figure 13:**
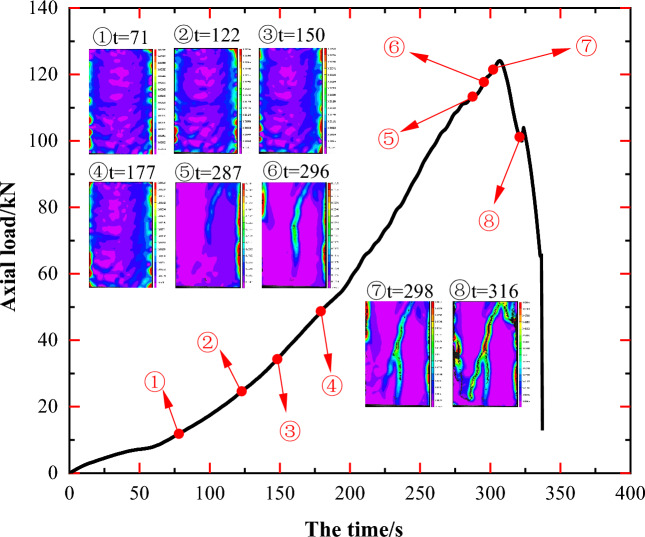
Principal strain state at some moments on the surface of JL5 specimen.

**Figure 14:**
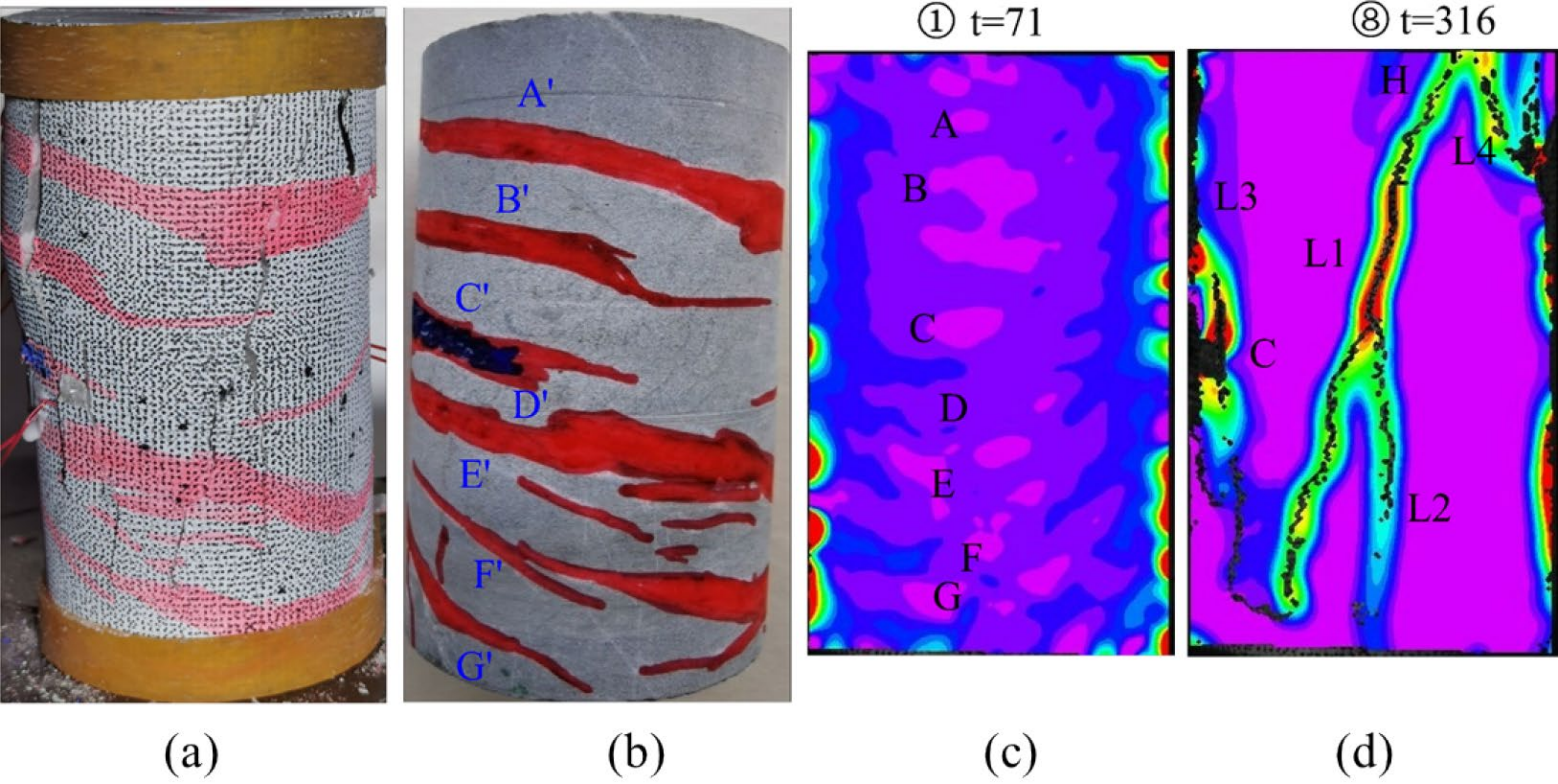
Analysis of the state of the JL5 specimen before and after the test and the main strain during the test: (**a**) Final state of the specimen, (**b**) Initial state of the specimen, (**c**) Principal strain of the specimen at t = 71 s, (**d**) Principal strain of the specimen at t = 316 s.

For specimen JL5, as depicted in Fig. 13 during steps ①–⑤, the stress concentration in the middle of the specimen is not overt, yet it can be observed that during these steps, especially in step ① as illustrated in Fig. 14(c), the principal strain values at locations A–G are notably larger than those in the surrounding areas. In step ①, the maximum value of the principal strain at these locations is approximately 0.0011 mm, whereas the maximum value of the principal strain in the surrounding areas is approximately 0.0002 mm. Close examination of these locations with significant principal strains reveals a resemblance to the positions of the main calcite veins A'-G' shown in Fig. 14b. This alignment can be attributed to the likelihood of stress concentration occurring at the original calcite veins of the specimen.

Upon combining the final damage state of the specimen depicted in Fig. 14a with the specimen's condition at t = 316 s as shown in the principal strain diagram (Fig. 14d), it becomes evident that as the experiment progressed, a stress concentration region emerged at location H on the upper right side of specimen JL5. Ultimately, within this region, in addition to the formation of the longitudinal crack L1 and its branch L2, a new crack, L4, developed. Simultaneously, on the left side of specimen JL5, another crack, L3, originated from the top. Consequently, the specimen exhibited a total of four distinct main cracks, evolving along the longitudinal direction. Upon scrutinizing the initiation locations and penetration paths of these four main cracks, there was no apparent overlap with the calcite veins; instead, the main cracks primarily intersected with the calcite veins.

After analyzing the main strain development and crack extension penetration paths during the tests of the aforementioned five specimens, the following conclusions can be drawn. In essence, the impact of calcite-filled veins on the fracture path is dependent on their alignment, particularly when they are parallel or nearly parallel to the loading direction. The fracture path is inherently influenced by the loading conditions, and these veins play a facilitating role in the cracking process when aligned in parallel with the loading direction.

1. The laterally distributed calcite veins (e.g., the structural faces laterally distributed in specimens JL2, JL3, JL4, and JL5) often induce areas of stress concentration in the rock mass at the onset of loading, but these stress concentration areas ultimately do not lead to transverse cracking in those regions of the specimens. As previously highlighted, calcite-filled veins serve as vulnerable surfaces. Initially, during loading, transverse veins experience compression, causing stress concentration in these areas. Since these veins are transverse, no cracks form parallel to them. However, as the crack initiates, a shift occurs, with increased energy directed towards crack expansion. Consequently, stress concentration diminishes and disperses in these regions. This phenomenon is illustrated in Fig. 10, where, at point 3, the initiation of cracking on the sample's top surface results in the dissipation of stress concentration at point A.
2. When calcite veins are distributed longitudinally (e.g., the longitudinally distributed calcite veins in specimens JL1, JL2, JL3, and JL4), it often results in the initiation of the specimen's main crack coinciding with the end points of the calcite veins, and the expansion path of the main crack is also influenced by the orientation of the calcite veins. In summary, the impact of calcite-filled veins on the fracture path is contingent upon their alignment, particularly when they are parallel or nearly parallel to the loading direction. The fracture path is inherently influenced by the nature of the loading, with these veins playing a facilitating role in the cracking process when aligned in parallel with the loading direction.

**Figure 15:**
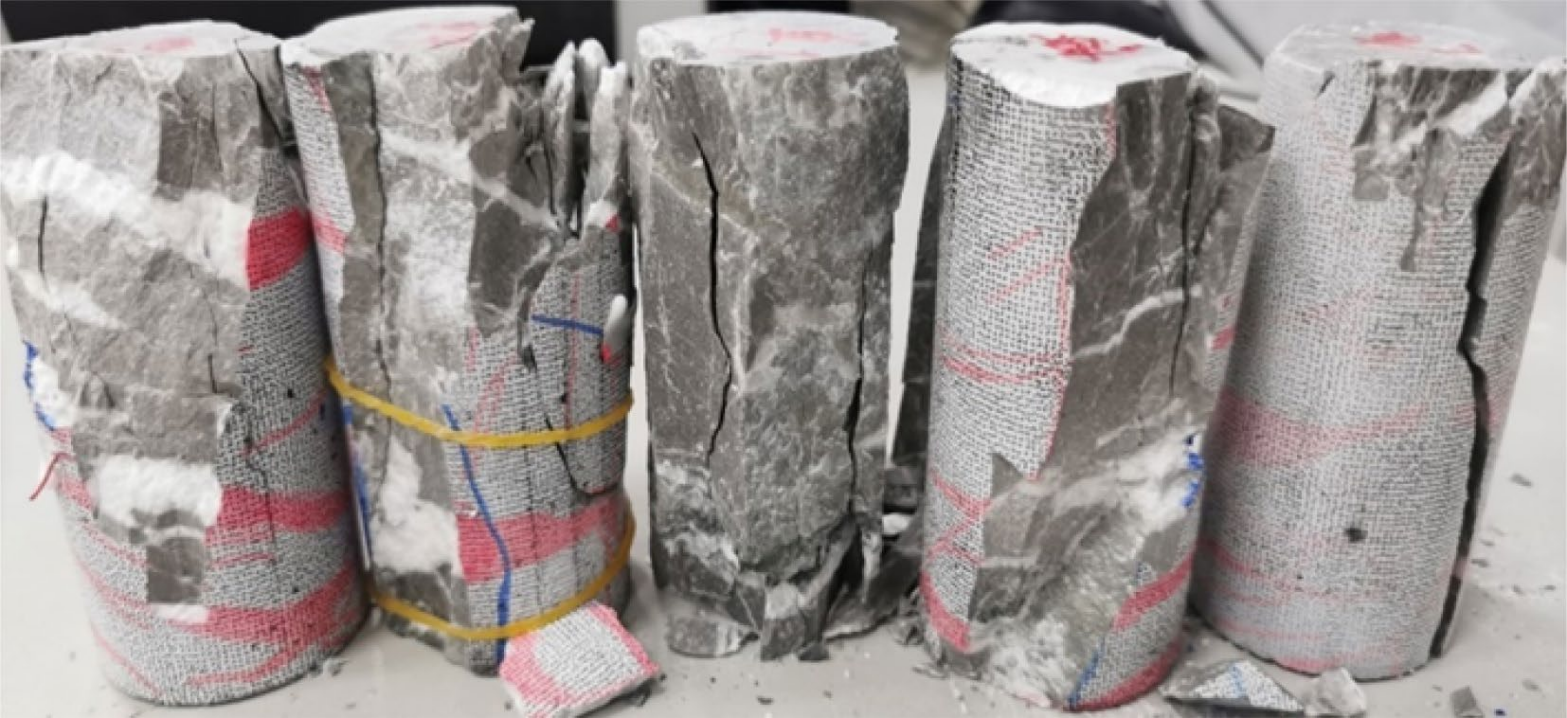
Damage state of all specimens after uniaxial test.

The analysis above focuses solely on the alterations in the main strain and the evolution of cracks in the orthogonal plane (the plane examined by the 2D DIC system) of the specimen during the uniaxial compression test. The overall final damage state of the specimen is depicted in Fig. 15.

Upon observing the overall damage state of the specimens post-test, it is evident that, despite sharing the same composition in both the main body and calcite veins, each specimen exhibits a distinct crack expansion pattern during the uniaxial compression test under nearly identical experimental conditions. This variation may arise from differences in the distribution states and contents of calcite within the specimens. Consequently, the presence of calcite veins induces divergence in the crack expansion, penetration paths, and overall damage pattern among the specimens. Notably, the damage pattern is more significantly influenced by longitudinally distributed calcite veins. Cracks induced by axial loading primarily result from the influence of these veins, extending along them in multiple sections. The penetration paths are minimally affected by transversely distributed calcite veins, as these cracks traverse the interior of these veins, penetrating longitudinally through the specimen. Ultimately, the specimen undergoes damage due to the longitudinal penetration of the generated cracks. The damage pattern observed in these specimens aligns with the general trend of uniaxial rock damage, manifesting as either monoclinic shear damage or axial splitting damage.

### The effect of calcite veins on the strength of the specimens

**Figure 16:**
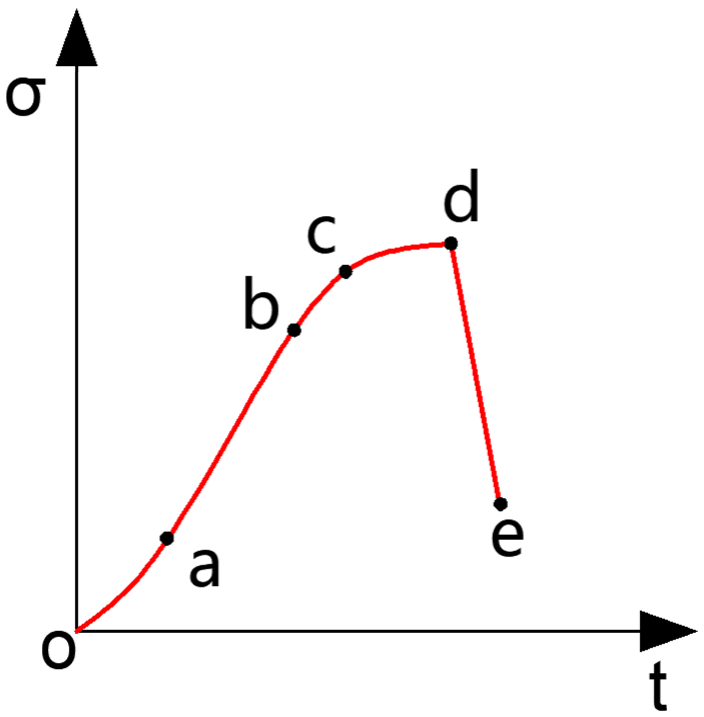
Strength curve of uniaxial compression test of conventional rock.

The strength curve from a conventional uniaxial compression test on rock is illustrated in Fig. 16. The entire damage process of the rock specimen can be categorically delineated into five stages: (a) Stage oa: Microgap compression and density stage. The curve in this section exhibits an upper concave shape, with a gradually increasing slope. (b) Stage ab: Elastic deformation stage. The line takes on an almost straight form as the micro gap in the specimen further closes and compacts, compressing voids. (c) Stage bc: Initial expansion stage. The curve transitions from the straight form of the ab section to a slightly convex shape, signifying a shift from elastic deformation to the destructive stage. Cracks within the specimen start to develop during this stage. (d) Stage cd: Destruction stage. Compared to the bc section, the curve rise decelerates, becoming more convex. Specimen expansion accelerates, and deformation increases rapidly. (e) Stage de: Deformation and destruction stage after the peak. The curve in this section exhibits a rapid decline, indicating sharp specimen damage, with a sudden release of energy^29,30^.

To validate the conformity of the load curves obtained from uniaxial compression tests on rock specimens featuring natural calcite veins in this study with the load curves of conventional rock specimens as described earlier, it is imperative to scrutinize whether their overall characteristics and the various segments of the curves (oa segment, ab segment, bc segment, cd segment, de segment) align with those of conventional rock load curves. Within this study, an analysis was conducted on three AE characteristic parameters, namely impact number, cumulative impact number, and amplitude. This analysis aimed to identify six characteristic points (o, a-e) on the time-axis load curve for each specimen.

**Figure 17:**
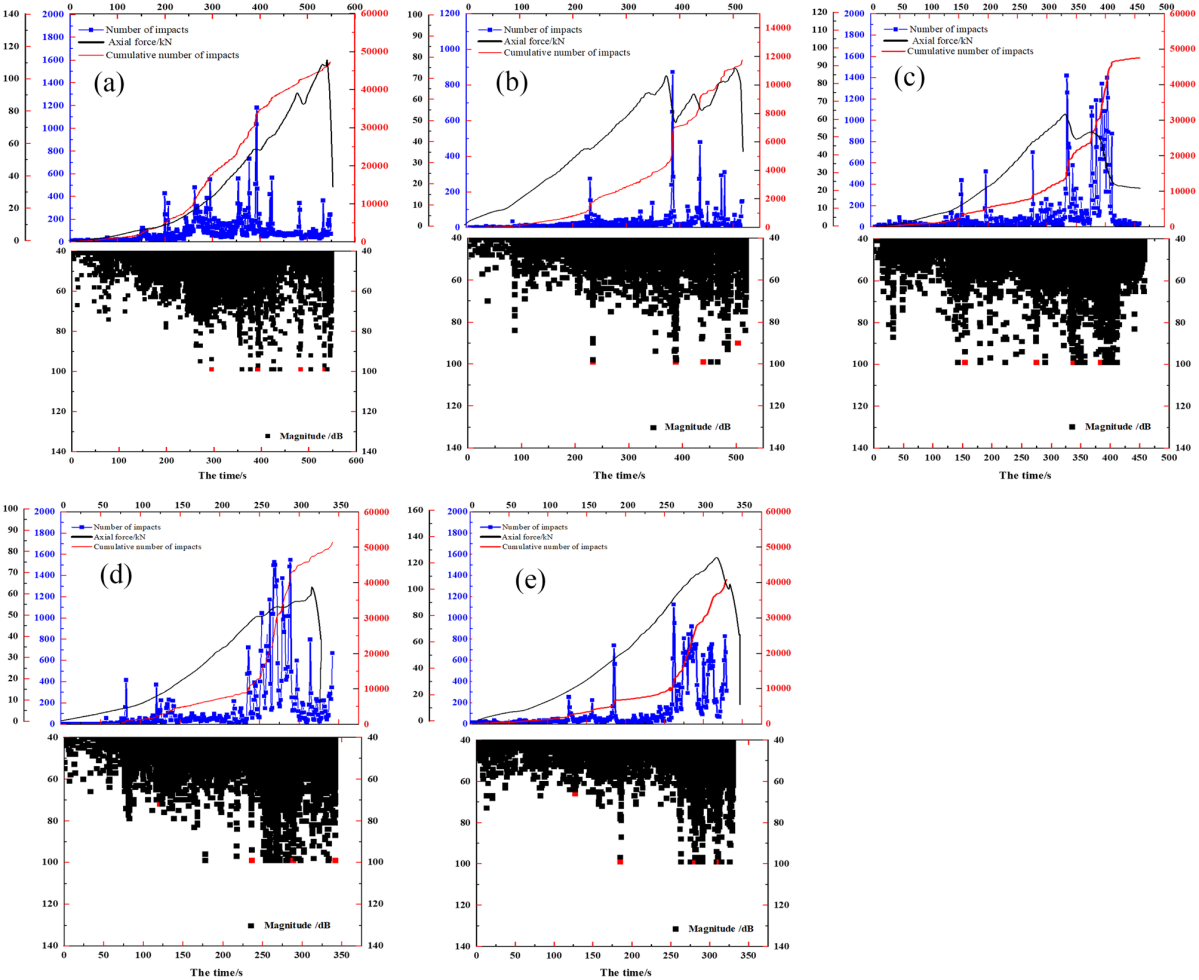
Curves of the number of impacts, cumulative number of impacts, amplitude, and axial load of each specimen with time: (**a**) JL1 (**b**) JL2 (**c**) JL3 (**d**) JL4 (**e**) JL5.

The curves depicting impact number, cumulative impact number, amplitude, and axial load over time for each specimen are illustrated in Fig. 17. As depicted in Fig. 17a, the curves and data for the AE characteristic values and the variation of axial load over time for the JL1 specimen indicate that, from t = 0 s to t = 295 s, both the axial load-time curve and the cumulative impact number-time curve exhibit an upward-concave shape. During this period, the slopes of both curves gradually increase. Subsequently, from t = 295 s to t = 393 s, the slope of the curve tends to stabilize, approximating a straight line. At t = 393 s, 483 s, and 534 s, the axial loads for all rock specimens exhibit extreme values, aligning with extreme values in amplitude and impact number at those specific moments. Therefore, by associating the peak points or inflection points of each curve in Fig. 17a, the corresponding times for these characteristic points on the load-time curve (a–e) for specimen JL1 are identified as t = 295 s, t = 393 s, t = 483 s, t = 534 s, and t = 553 s, respectively. Using a similar methodology, the times corresponding to characteristic points (a–e) on the time-axial load curves for all five specimens can be conclusively determined, as shown in Table 3.

**Table 3:** Time corresponding to different characteristic points in the time-axial load curve for five specimens.

Number of the specimen	The time of each feature point /s				
	a	b	c	d	e
JL1	295	393	483	534	553
JL2	232	387	438	513	517
JL3	155	276	334	384	458
JL4	121	237	289	341	342
JL5	127	185	285	311	330

It is essential to highlight that the axial load-time curves for specimens JL2 and JL3 deviate from the typical characteristics observed in conventional uniaxial compression tests on rocks within certain time intervals. For instance, at t = 387 s and t = 438 s, the JL2 curve experiences a sudden decline due to an abrupt change in the axial load value. During the period from t = 387 s to t = 438 s, the curve fails to exhibit a steady rise, deviating from the typical characteristics of the axial force–time curve in the bc section of conventional rock uniaxial compression tests. However, the analysis of intervals 0–232 s, 232–387 s, 387–438 s, and 438–513 s still aligns with the characteristics of the axial force–time curve in the oa, ab, bc, and cd segments of conventional rock triaxial compression tests. Consequently, t = 387 s and t = 438 s are still considered as the time nodes corresponding to feature points b and c, respectively.

Similarly, the axial load of specimen JL3 at t = 384 s does not represent the maximum value for the entire curve. In the time frame from t = 334 s to t = 384 s, the axial load of the specimen experiences a gradual decrease instead of a progressive increase. However, a comprehensive analysis of the curve characteristics within intervals 0–155 s, 155–276 s, and 276–334 s reveals consistency with the characteristics within the oa, ab, and bc zones, respectively. Thus, t = 384 s is still regarded as the time node corresponding to characteristic point d.

In the case of specimens JL1, JL4, and JL5, their axial load-time curves align more closely with the typical development pattern of axial force–time curves observed in conventional rock uniaxial compression tests. Clear identification of the five characteristic points a-e is evident in these curves. This suggests that the macroscopic load-time curves of the aforementioned five rock specimens, which contain natural calcite veins, broadly adhere to the axial force–time curves of conventional uniaxial compression tests on rocks. Upon comparing the analysis with the AE eigenvalue curves, all specimens successfully exhibit the identification of the five characteristic points a-e on their load-time curves. Consequently, the presence of calcite veins does not significantly alter the development pattern of the load curves or the damage pattern of the specimens. The overall load curves for each specimen still adhere to the principle of gradually rising to the peak point before rapidly declining. The entire damage process for each specimen still predominantly encompasses five stages: oa section-microgap compacting stage, ab section-elastic deformation stage, bc section-initial expansion stage, cd section-destruction stage, and de section-post-peak deformation and destruction stage.

**Figure 18:**
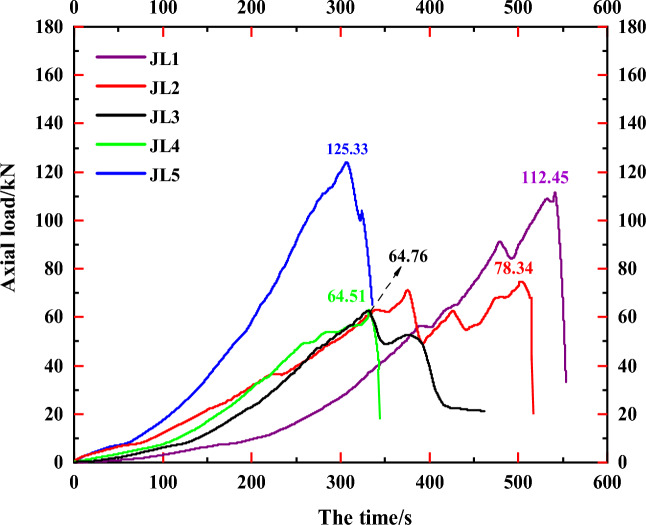
Load-time curves of specimens.

Nevertheless, despite conducting uniaxial compression tests on five specimens with comparable dimensions, identical main rock body composition, and nearly identical experimental conditions, the final load-time curves exhibited notable variations in values, as illustrated in Fig. 18. For instance, their peak axial loads were recorded as 112.45 kN, 78.34 kN, 64.76 kN, 64.51 kN, and 125.33 kN, respectively. Notably, the load curves of specimens JL2 and JL3 deviated from the characteristic patterns observed in conventional rock load curves within specific zones. These disparities suggest that the presence of calcite veins still influences strength characteristics, such as the uniaxial compressive capacity of the rock. This influence may stem from variations in the distribution patterns and contents of calcite within the specimens.

In conclusion, the presence of calcite veins did not result in a substantial alteration in the damage pattern observed during uniaxial compression tests on the specimens, nor did it significantly impact the temporal development pattern of axial load. However, it did exert an influence on the overall uniaxial compression strength of the rock mass. Here, some explanations are needed for the content of this study: since the uniaxial compression tests of the five specimens were carried out under the same test conditions, the specimens selected were from the same rock mass, the main rock portion of the five specimens was limestone, and the veins were all calcite, the differences in the test results were mainly due to the irregularity of the veins in the specimens. This difference may be related to the thickness, strike, and content of the veins. Since the five specimens in this study were obtained from natural rock bodies, the strike and thickness of the veins in each specimen varied markedly and did not follow a similar pattern. It is more difficult to determine the effect of a single element, such as the orientation of the veins, on the uniaxial compressive strength and damage pattern of the rock specimens by controlling the form of a single variable. Therefore, for the sake of the rigor of the results, this study only clarified the relationship between the direction of the main cracks of limestone and the overall distribution mode (transverse or longitudinal) of its internal calcite veins, and proved that the strength characteristics of the rock mass are significantly influenced by its internal calcite veins. Studies on the influence of vein distribution patterns, thickness and strength on the mechanical properties of the rock mass are not included. These will be investigated in the course of subsequent studies with rock-like materials.

## Conclusion

The findings from the aforementioned studies have been synthesized to draw the following conclusions:

1. Calcite veins in limestone significantly influence its mechanical behavior and fracture extension: (a) Laterally distributed calcite veins often induce stress concentration in the rock mass at the onset of loading. However, these areas of stress concentration do not ultimately lead to transverse cracking in the specimens; (b) Longitudinally distributed calcite veins often result in the initiation of the main crack coinciding with the end points of the veins, influencing the expansion path of the main crack; (c) Longitudinal calcite veins impact the path of crack expansion but do not cause significant alterations in the final damage form, maintaining alignment with conventional rock mass damage under uniaxial compression.
2. The presence of calcite veins affects the overall magnitude of uniaxial compressive strength in the rock mass: Despite nearly identical test conditions for five specimens, variations in their final peak axial loads were observed, attributed to differences in the distribution and content of calcite veins within each specimen. It is noteworthy that while the presence of calcite veins influenced the uniaxial compressive strength, the overall axial load-time development pattern adhered to the conventional pattern observed in uniaxial compression tests on rocks, characterized by the distinct stages Oa, ab, bc, cd, and de.

## Data Availability

The datasets used and/or analysed during the current study available from the corresponding author on reasonable request.
